# Insights from Shigella bacteriophage genomes analysis

**DOI:** 10.6026/9732063002002050

**Published:** 2024-12-31

**Authors:** Pratanu Kayet, Surajit Bhattacharjee, Shanta Dutta, Surajit Basak

**Affiliations:** 1 Division of Bioinformatics, ICMR-National Institute for Research in Bacterial Infections, Kolkata, India; 2 Department of Molecular Biology and Bioinformatics, Tripura University, Suryamani nagar-799022, Tripura, India; 3 Division of Bacteriology, ICMR-National Institute for Research in Bacterial Infections, Kolkata, India

**Keywords:** Anti-CRISPR, shigella phage, functional category, tRNA, antimicrobial resistance (AMR)

## Abstract

Shigella species, a major cause of shigellosis, remain a substantial global health issue and the emergence of antibiotic-resistant Shigella strains has aggravated the situation. Hence, four Shigella phages were investigated to provide insights into the evolutionary trajectories and genomic properties of Shigella-infecting bacteriophages using comparative genome analysis. Analysis shows that these four phages belong to the Tequatrovirus genus and include a considerable number of proteins for 'Tail' and "DNA, RNA and Nucleotide Metabolism," indicating their aptitude for specialized host interaction and replication efficiency. The identification of 10 tRNAs further support that, these phages have high replication efficiency. Thus, this study improves our understanding of phage evolution by exposing the genetic mechanisms that drive phage adaptability and host specificity. This also highlights the significance of phage genomic research in developing viable therapies for antibiotic-resistant Shigella infections.

## Background:

Shigella species are facultative intracellular, gram-negative bacteria that cause shigellosis, a highly contagious illness that is mostly characterized by acute gastroenteritis and diarrhoea [1], with more than 165 million cases and around 1 million fatalities every year. Shigella continues to pose a serious threat to world health, especially in low- and middle-income nations [2, 3]. Aside from its toxicity, Shigella's development of antibiotic-resistant strains has grown in importance as a public health issue, making treatment plans more difficult and emphasizing the need for alternate therapeutic approaches [4]. The use of viruses that specifically infect and destroy bacteria, known as bacteriophage treatment, has drawn a lot of attention as potential tactic to counteract Shigella strains that are resistant to multiple drugs [5-6]. Bacteriophages, often known as phage's, are a diverse and widespread group of viruses that highly selectively infect bacteria [7]. For a variety of bacterial illnesses, including those brought on by pathogens resistant to antibiotics like Shigella, they have been investigated as possible therapeutic agents [8- 9]. Targeting harmful bacteria without altering the human microbiome is one of phage therapy's many benefits, which makes it a good option when antibiotics haven't worked [10- 11]. The ability of Shigella-infecting phages to function as efficient bio-control agents has been demonstrated by recent research, particularly in light of the developing issue of Shigella's resistance to several antibiotic classes [12, 13]. Shigella phages have not yet reached their full therapeutic potential despite these encouraging uses, mainly because a better understanding of their genetic diversity, evolutionary trajectories and interactions with bacterial hosts [14]. Similar to their bacterial hosts, phages are influenced by evolutionary forces like bacterial resistance mechanisms and phage counter-defenses influence the co-evolutionary dynamics between Shigella and its infecting phages [15]. Shigella bacteria have evolved a number of defense mechanisms, including as receptor alterations, the acquisition of defense systems like CRISPER-Cas and the synthesis of anti-phage enzymes, to avoid phage invasion [16]. Phages respond by developing new strategies to get past these bacterial defenses, which leads to a continuous evolutionary "arms race." This co-evolution process is essential for determining the phages' long-term efficacy as therapeutic agents as well as for comprehending their biological success in natural settings [17]. The study of phage evolution is essential for the creation of phage-based treatments because of the dynamic nature of this interaction, which forces phages to continuously change in order to overcome novel bacterial resistance tactics [18, 19]. By investigating the genetic mechanisms that drive phage adaptability, we can find factors that influence phage efficacy, host specificity and resistance to bacterial defense systems [20]. Phages that evolve to escape bacterial CRISPR-Cas systems or other immune mechanisms may provide an extra benefit in phage therapy by allowing phages to infect and proliferate even in the presence of bacterial resistance [21]. Shigella phages, like all other bacteriophages, have a limited host range, infecting just certain strains of Shigella, whilst others have a broader host spectrum and these host specificity differences are shaped by the development of important genomic characteristics that govern phage-host interactions, such as the proteins involved in host recognition and genome injection, so comparative genomic studies of Shigella phages are thus critical for discovering conserved genetic components that may be significant for phage infectivity, as well as understanding the processes that contribute to phage population diversity [22]. The application of comparative genomics to phage evolution enables researchers to find evolutionary patterns that provide insights into how shigella phages adapt to their bacterial hosts. This genomic perspective also helps to explain how shigella phages evolve in the face of selective pressures from bacterial resistance mechanisms and immunological responses including the CRISPER-Cas system [23]. Some phages create anti-CRISPR proteins that disrupt bacteria's CRISPER-Cas defensive system, allowing the phage to successfully infect the host despite bacterial defenses. The discovery and characterization of anti-CRISPR genes in Shigella phages may provide important insights into how phages develop to overcome bacterial resistance. Furthermore, the presence of antimicrobial resistance (AMR) genes in phage genomes may suggest that phages have been exposed to antibiotic-resistant bacterial strains and it may even contribute to the horizontal transmission of resistance genes among bacteria [24, 25- 26]. Therefore, it is of interest to show that Shigella phage evolution is critical for enhancing our knowledge on phage therapy and better understanding of phage-bacteria interactions through comparative genomics analysis of Shigella-infecting bacteriophages.

## Methodology:

## Sequencing and assembly:

## Isolation and quantitative analysis of DNA:

The four bacteriophages (ADG1, CDR3, AKR2 and TMC4) were isolated from lake water in Kolkata, India. The lake was chosen as a sampling site because it is exposed to both natural microbial ecosystems and probable human or animal waste, both of which are known repositories of phages infecting intestinal diseases Shigella. Following the initial screening and isolation processes, only these four phages were successfully recovered and propagated for further research. Isolating bacteriophages infecting Shigella from an environmental source is consistent with our study's purpose of investigating naturally existing phage diversity and its possible involvement in countering antibiotic-resistant Shigella strains. Their availability as viable isolates from the sampling effort made them ideal candidates for genetic analysis. These phages give an important picture of the genetic diversity, functional adaptations and evolutionary dynamics of Shigella-specific phages from an environmental reservoir. By characterizing these phages, we hope to provide light on their genomic features, host interaction mechanisms and therapeutic potential, as well as provide insights into the larger ecological and evolutionary background of Shigella-infecting phages. Samples were processed using CTAB DNA isolation method. DNA quantity was measured using Qubit® 4.0-fluorometer and DNA quality was analyzed on 1.0% agarose gel.

## Preparation of library:

The paired-end sequencing library was prepared using Twist NGS Library Preparation Kits for Illumina® (CAT No. ID 104119). The library preparation process was initiated with 50 ng input. DNA was enzymatically sheared into smaller fragments by kit protocol and continuous step of end-repair and A-tailing where an 'A' is added to the 3' ends making the DNA fragments ready for adapter ligation. Following this step, illumine specific adapters are ligated to both ends of the DNA fragments. These adapters contain sequences essential for binding barcoded libraries to a flow cell for sequencing, allowing for PCR amplification of adapter-ligated fragments and binding standard Illumina sequencing primers. To ensure maximum yields from limited amounts of starting material, a high-fidelity amplification step was performed using HiFi PCR Master Mix.

## Quantity and quality check (QC) of library on agilent tape station 4150:

The amplified libraries were analyzed on TapeStation 4150 (Agilent Technologies) using High Sensitivity D1000 ScreenTape® as per manufacturer's instructions.

## Cluster generation and sequencing:

After obtaining the Qubit concentration for the library and the mean peak size from Tape Station profile, library will be loaded onto illumina Novaseq 6000 for cluster generation and sequencing. Paired-End sequencing allows the template fragments to be sequenced in both the forward and reverse directions. The library molecules will bind to complementary adapter oligos on paired-end flow cell. The adapters are designed to allow selective cleavage of the forward strands after re-synthesis of the reverse strand during sequencing. The copied reverse strand is then used to sequence from the opposite end of the fragment.

## Genomic assembly and analysis:

After Gathering raw sequencing readings from a sequencer is the initial stage of our study. We use FastQC [27], a program that evaluates quality of raw reads. As low-quality readings might induce biases or inaccuracies in later studies, this quality check is essential. After quality control, we trim any low-quality areas from the reads and eliminate adapter sequences using Trimmomatic [28]. This reduces the possibility of inaccurate data and raises the assembly's overall correctness by guaranteeing that only high-quality sequences are used for downstream assembly. After that, we go on to de-novo assembly, which uses the trimmed reads to rebuild the genomes without a reference genome. We make use of four distinct assemblers: Megahit [29], Velvet [30], SPAdes [31] and SKESA [32]. The benefit of comparing several assemblies to choose the most accurate one is that each of these assemblers uses distinct genome assembly algorithms and techniques. In our case, SKESA produced the assembly with the highest N50, making it the best option for additional analysis. The resulting assemblies are evaluated for quality using QUAST [33], a tool that assesses multiple metrics, including the N50 value, which is crucial for determining the completeness of the assembly. Following the selection of the assembly, Prokka [34] is used for genome annotation, making predictions about the genes and proteins present in the phage genome. Prokka provides a thorough summary of the phage's gene composition and functional potential by identifying coding sequences, tRNAs and other genomic characteristics.

## The GenBank accession numbers for four bacteriophage genome sequences are:

ADG1: PQ666539

AKR2: PQ666540

CRD3: PQ666541

TMC4:PQ666542

## Functional classification:

We use the PHROGs database [35], an extensive resource that lists phage proteins and their functional annotations, to categorize the discovered proteins' functional roles. We find the best matches for each of our proteins by comparing our phage proteins with the PHROGS database using Blastp [36]. We use the Galaxy server's blast best Hit Identification Program to make sure we choose the most accurate functional classification. By using sequence similarity, this program lets us sift through the BLAST findings and find the most pertinent hits. Following the identification of the best matches, we categorize the proteins according to their matching PHROGs IDs, which offer information about the proteins' putative functional roles in relation to the phage's lifecycle, host interactions and other biological processes.

## Core genome analysis:

Using Roary [37], a program intended to examine the variety of bacterial and viral genomes, we conduct pan-genome analysis after acquiring the gene annotation file from Prokka. We can identify the core genome the genes that all phages share and the accessory genome the genes that are found in some but not all phages with the aid of Roary's pan-genome analysis. In order to assess the genomic similarities and differences among the phages and get insight into their evolutionary history and functional variety, it is critical to comprehend the distribution of these genes. Additionally, this study aids in the identification of distinct genes that might be involved in particular traits, including virulence or host specificity.

## Multivariate analysis:

For correspondence analysis we use CodonW [38], a program that enables us to examine Amino Acid Usage (AAU) patterns, to conduct multivariate analysis in order to investigate the evolutionary dynamics of the phages. CodonW looks for any notable variations or patterns in the amino acid composition of the genes across the phages. Since changes in amino acid utilization can reveal selection pressure, functional adaptability, or evolutionary restrictions acting on the phages, this approach is useful for identifying evolutionary patterns in the genome. We can learn more about the evolutionary forces that have influenced the phages' genetic composition by contrasting these trends between the core and auxiliary genomes.

## Whole genome phylogenetic tree:

We create a full genome phylogenetic tree to comprehend the evolutionary relationships between our phages and other closely related phages. Using the NCBI database, which has a sizable number of bacteriophage genomes (as of October 2024), we start by running a BLAST search. A subset of 83 closely related phages is obtained by applying strict criteria to identify phages with at least 90% query coverage and 95% sequence identity. MAFFT, a tool that uses complex algorithms to provide precise multiple sequence alignments, is used to align the chosen genomes. RAxML [39], which is based on the GTR + G + I evolutionary model, is then used to build a maximum likelihood phylogenetic tree using the alignments. This model provides a strong and trustworthy phylogenetic tree that shows the evolutionary relationships among the phages and places them in the larger context of other phages in the NCBI database by taking into consideration the substitution rates and variability throughout the genome.

## Anti-CRISPR and AMR gene identification:

The existence of anti-CRISPR and antimicrobial resistance (AMR) genes in phage genomes is a critical topic of research since these genes can influence the phages' capacity to avoid host immune systems and their possible role in antimicrobial resistance. To identify these genes, we use the AcrDB [40] and Anti-CRISPRdb [41] databases for anti-CRISPR gene sequences, as well as the CARD database [42] for AMR genes. We use BLASTP to match the anti-CRISPR and AMR gene sequences in these databases with the proteins from our phage genomes. In order to comprehend how the phages interact with bacterial hosts and contribute to the dynamics of antimicrobial resistance, it is essential that we discover any potential anti-CRISPR or AMR genes in our phages. The identification of these genes can also aid in assessing the phages' possible application in therapeutic contexts, where resistance gene modification and bacterial immune system evasion are crucial elements.

## Results:

## Comparative genomic analysis:

**Figure 1:**
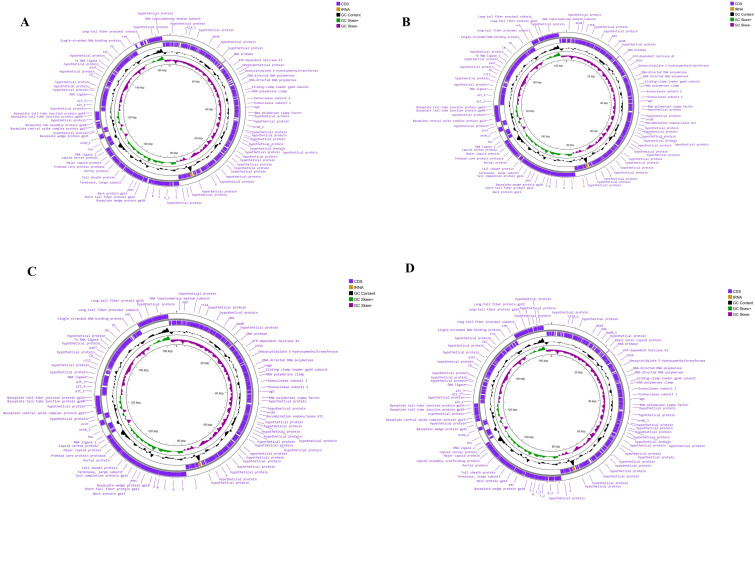
Genomic map of four phages, where purple colored lines are CDS and yellow colored lines are tRNA. Where A, B, C, D is representing the phage ADG1, AKR2, CRD3 and TMC4 respectively.

The genomes of phages ADG1, CDR3, AKR2 and TMC4 have been sequenced and uploaded as supplementary files. The GenBank accession numbers are also provided in the 'Methodology' section. These phages have the following genome lengths: ADG1 (164,945 bp), CDR3 (165,220 bp), AKR2 (165,034 bp) and TMC4 (164,971 bp). Their average G+C content are: 35.16%, 35.52%, 35.49% and 35.35%, respectively. Figure 1 shows a schematic genomic map of the four phages developed using the Proksee server [43]. In this map, a visual representation of the genomic structures is provided where the inner ring represents coding sequences (CDS) in blue, whereas tRNA genes are highlighted in pink (Figure 1). An analysis of annotated open reading frames (ORFs) of the four phages indicates that the phage ADG1 encodes 285 proteins, CDR3 encodes 255 proteins, AKR2 encodes 254 proteins and TMC4 encodes 291 proteins. These include structural proteins, genome packaging proteins, lysis proteins, holins and tail proteins. Each of the phage genomes has 10 tRNA genes: tRNA-Arg(tct), tRNA-Asn(gtt), tRNA-Tyr(gta), tRNA-Met(cat), tRNA-Thr(tgt), tRNA-Ser(tga), tRNA-Pro(tgg), tRNA-Gly(tcc), tRNA-Leu(taa) and tRNA-Gln(ttg). These tRNAs are thought to give a high level of independence from the host's translational machinery, which is a well-known approach for improving phage protein production during infection [44]. Taxonomic classification put all four phages to the Tunavirus genus, suggesting their evolutionary link within this group. These findings show the four phages' share strong evolutionary relationship and common genomic characteristics, providing vital information for the study of bacteriophage genetics and taxonomy.

## Functional classification:

Functional classification divides phage proteins into nine categories based on their biological functions (Table 1). However, we did not consider two functional categories namely, 'Unknown function' and 'Others' in our analysis. The majority of phage proteins belong to the "Tail" and "DNA, RNA and Nucleotide Metabolism" categories (Table 1), indicating their role of the phage interaction with its host and replication inside the host. Phage tails are crucial for host recognition, attachment and genome injection, particularly in tailed bacteriophages and a proteins involved in DNA, RNA and Nucleotide Metabolism are essential for transcription as well as replication of the virus's genome in the host cell.

## Correspondence analysis on amino acid usage of Shigella bacteriophage:

**Figure 2:**
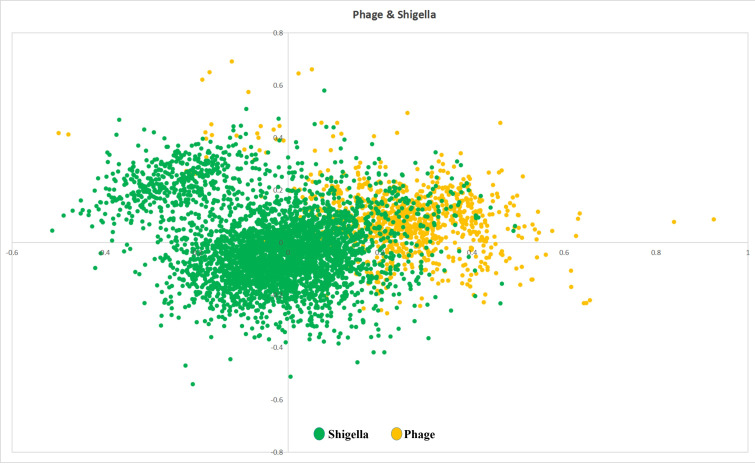
Amino acid usage of shigella bacterium with one of the four bacteriophages. Green colored points represent Shigella and yellow colored points represent Phage.

**Figure 3:**
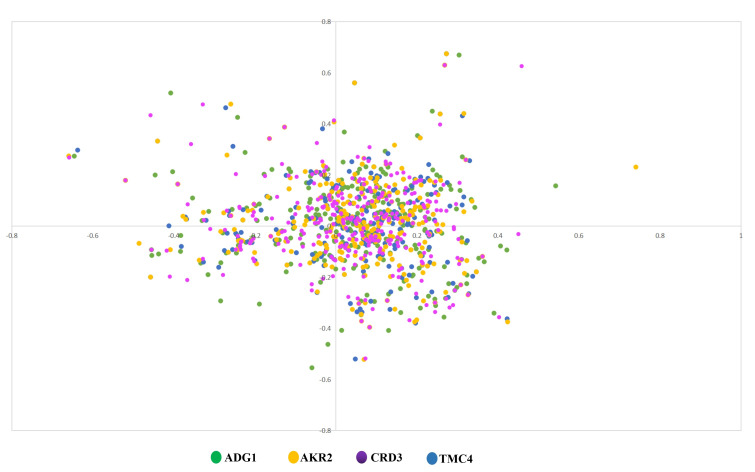
Similarity in amino acid usage of four bacteriophages. Green, yellow, purple and blue colored points represent each of the four phages

We performed Correspondence analysis on amino acid usage of the four newly sequenced Shigella bacteriophage genomes taken together to ascertain if there exists any difference in amino acid usage among the four bacteriophages. Figure 2 clearly shows that genes from four bacteriophages are completely overlapped on each other indicating identical amino acid usage of four bacteriophages. Later, we performed Correspondence analysis on amino acid usage by taking the one of the four bacteriophages and its host, (*i.e.,*) Shigella flexneri. Here, 10% of Shigella flexneri genes are overlapping with the 17.5% of bacteriophage genes (Figure 3). More than 80% of the preferred amino acids are perfectly matching between the bacteriophage and its host.

## Whole genome tree and core genome construction:

**Figure 4:**
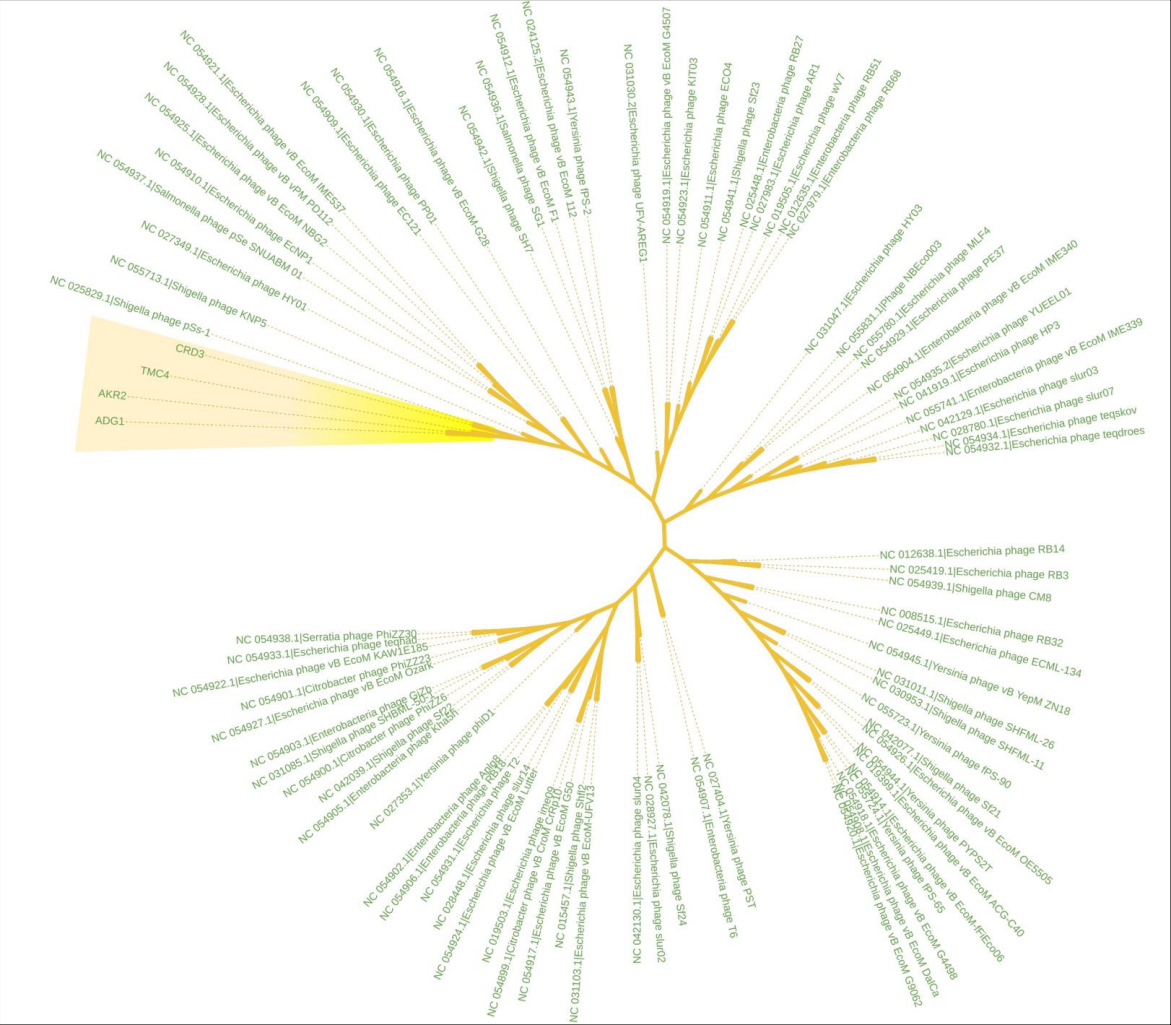
Phylogenetic tree of isolated four phages with other phage species. The four isolated phages are highlighted with yellow gradient box.

A complete genome tree (Figure 4) demonstrates a strong evolutionary link among the phages, particularly with Shigella and Escherichia phages. Shigella phage KNP5 and Shigella phage pSs-1 have been observed to be the closest relative with the four newly sequenced phages. This observation highlights the phages' shared evolutionary history and genetic similarities. We also built core genomes from the newly sequenced four phages. This core genome provides a unified framework of conserved genetic components throughout the bacteriophages studied. The core genome also depicts similar amino acid usage patterns among the four phages, indicating similarity of amino acid composition in the core genome.

## Anti-CRISPR and AMR gene identification:

We identified Rz-like spanin, belong to the lysis functional category and the SbcC-like subunit of palindrome-specific endonuclease and belong to DNA, RNA and nucleotide metabolism. Both proteins have the ability to demonstrate anti-CRISPR activity, implying that they are involved in countering host CRISPR-Cas systems during infections. Additionally, we also searched for antimicrobial resistance (AMR) genes in these phages and found one protein called dihydrofolate reductase, belong to the DNA, RNA and nucleotide metabolism group. Further analysis revealed that this protein is orthologous to the known AMR proteins dfrA9, dfrA10 and dfrA26, with more than 70% sequence identity. These findings indicate that, while this protein may serve a natural role in phage biology, its similarity to AMR proteins warrants further investigation into its possible impact.

## Discussion:

The four phages show high genetic conservation, particularly with respect to important functional and structural proteins. These phages also demonstrate distinct adaptive strategies to represent the dynamic interaction between phages and their bacterial hosts as is evident from correspondence analysis on amino acid usage. The four phages have a similar genome length (~165 kb) and G+C content (~35%), which is consistent with Tunavirus phage features. This consistency highlights the evolutionary forces that drive genomic stability within Shigella-infecting phages. Structural proteins, such as head-tail adaptors, tail sheath proteins and receptor-binding proteins (RBPs), are highly conserved, implying common processes of host recognition, attachment and genome transport. For example, RBP is closely related to those found in Shigella phage JK23 and Escherichia phage BYEP02, highlighting the evolutionary requirement of preserving host receptor recognition [45]. Packaging proteins, such as the portal protein in our phages, share similarity with related phages, indicating a common role in effective DNA entry and departure during assembly and infection [46]. These conserved structural traits are consistent with previous research on Enterobacteriaceae phages, supporting the fact that these phages have evolved robust mechanisms to enable effective infection and multiplication in similar bacterial environments [47]. Beyond structural proteins, these phages have remarkable adaptive traits that improve infectivity and fitness. The presence of auxiliary metabolic genes, such as thymidylate synthase in our phages and dihydrofolate reductase in our phages, demonstrates how horizontal gene transfer (HGT) drives their evolution. These genes, which are essential for nucleotide metabolism, allow phages to avoid host metabolic limitations, resulting in faster replication. The identification of these genes highlights the significance of HGT in increasing phage-host compatibility, stressing bacteriophages' evolutionary flexibility in adapting to host metabolic pathways [48- 49]. Correspondence analysis of amino acid usage across the four newly sequenced Shigella bacteriophage genomes revealed perfect overlap in amino acid usage across all four bacteriophages (Figure 2), implying that their gene pools have substantially comparable signatures. This observation suggests that these phages may have common evolutionary origins or be subject to similar selective forces, resulting in convergent amino acid usage patterns. The lack of significant variation in amino acid usage among bacteriophages may possibly reflect functional constraints imposed by the need to properly infect and reproduce within Shigella hosts. When we broadened our study to look at the amino acid usage of one of the bacteriophages and its host, Shigella flexneri, we discovered a significant overlap between the two genomes (Figure 3). Specifically, 10% of the Shigella genes overlap with 17.5% of the bacteriophage genes, where mostly metabolic or structural genes are located. More than 80% of preferred amino acids are similar between the phage and its host supporting the hypothesis that phages and their bacterial hosts are co-evolving [50, 51]. This significant match in amino acid preferences may indicate that the phages are designed to interact well with host cellular machinery, implying a level of metabolic integration between the phage and host. Such integration may be required for successful phage multiplication and assembly within the host cell. These findings are consistent with the rising knowledge that bacteriophages are active players in bacterial evolution, potentially influencing host metabolism and gene transfer [52]. A notable finding is the anti-CRISPR proteins in each of the four phages that potentially play critical roles in the phage's evolutionary strategies and impact on the host. Rz-like spanin, belongs to the lysis functional category, indicates its significance in the phage's capacity to rupture host cell membranes. The SbcC-like subunit of a palindrome-specific endonuclease is involved in DNA, RNA and nucleotide metabolism, implying a more general regulatory role during the infection cycle. Both of these proteins are anti-CRISPR factors and could assist the phage bypassing the host's CRISPR-Cas defensive systems, increasing the phage's survival and multiplication inside the host. It shows that phages may evolve to circumvent bacterial immunity, influencing the dynamics of bacteria-virus interactions and perhaps facilitating horizontal gene transfer (HGT) between phages and their bacterial hosts [17]. We also found an antimicrobial resistance (AMR) gene in each of the four phages called dihydrofolate reductase (DHFR). This enzyme is highly similar to known AMR proteins including dfrA9, dfrA10 and dfrA26. The high sequence identity suggests that this protein may be involved in both the phage's fundamental metabolic processes and the host bacteria's AMR profile. Given DHFR's significance in folate metabolism and involvement in antimicrobial resistance mechanisms such as trimethoprim [53], the identification of this protein in Shigella phages raises crucial questions concerning its possible impact on the AMR evolution. It highlights the complex interplay between phages and their bacterial hosts, where phages may not only mediate the transfer of antimicrobial resistance genes but also act as potential vectors for the spread of resistance traits. Phylogenetic investigations show that these four phages share deep evolutionary ties, grouping them with other Shigella and Escherichia phages (Figure 4). It emphasizes their shared evolutionary origins and ecological niches, as well as the genetic traits shaped by common selective pressures. Core genome investigations emphasize the importance of critical proteins for genome replication, structural assembly and host interaction. These findings indicate that these phages developed from a common ancestor and adapted to specific hosts and environmental circumstances. The effect of HGT in altering phage genomes is most clear in these four phages, which has acquired auxiliary metabolic and structural genes that improve its ability to infect and reproduce within Shigella hosts. The findings of this work have important significance for both basic bacteriophage biology and applied phage therapy. Understanding the co-evolutionary dynamics of these phages and their hosts may potentially help to optimize their usage in combating antibiotic resistance. Future study should delve deeper into the functional roles of the identified proteins, investigate the ecological consequences of phage-host interactions and improve the usage of phages in combating the growing worldwide challenge of antibiotic resistance.

## Conclusions:

The amazing genetic conservation of critical structural and functional proteins, emphasizing the evolutionary mechanisms that maintain efficient host recognition, infection and reproduction is shown. Furthermore, the finding of anti-CRISPR proteins in phages provides an intriguing peek into the phage's capacity to elude bacterial immune systems for increasing its survival and proliferation within Shigella. Thus, this study provides a solid platform for harnessing the potential of phages in combating bacterial infections while addressing the complexities of microbial evolution and resistance.

## Figures and Tables

**Table 1:** Functional classification of phage proteins into nine categories based on their biological functions

**Query**	**phrog**	**category**
TMC4_169_head_closure_Shigella_phage_pSs-1	218	connector
TMC4_189_head-tail_adaptor_Ad2_Shigella_phage_pSs-1	211	connector
TMC4_191_head_closure_Hc2_Shigella_phage_pSs-1	679	connector
TMC4_188_head-tail_adaptor_Ad2_Shigella_phage_pSs-1	211	connector
TMC4_190_head_closure_Hc2_Shigella_phage_pSs-1	679	connector
TMC4_025_exonuclease_Shigella_phage_pSs-1	255	DNA, RNA and nucleotide metabolism
TMC4_044_nucleoside_triphosphate_pyrophosphohydrolase_Shigella_phage_pSs-1	173	DNA, RNA and nucleotide metabolism
TMC4_065_DNA_polymerase_processivity_factor_Shigella_phage_pSs-1	223	DNA, RNA and nucleotide metabolism
TMC4_252_dCMP_deaminase_Shigella_phage_pSs-1	174	DNA, RNA and nucleotide metabolism
TMC4_003_DNA_topoisomerase_II_Shigella_phage_pSs-1	551	DNA, RNA and nucleotide metabolism
TMC4_052_DnaB-like_replicative_helicase_Shigella_phage_pSs-1	19	DNA, RNA and nucleotide metabolism
TMC4_062_clamp_loader_of_DNA_polymerase_Shigella_phage_pSs-1	225	DNA, RNA and nucleotide metabolism
TMC4_089_anaerobic_ribonucleoside_reductase_large_subunit_Shigella_phage_pSs-1	4218	DNA, RNA and nucleotide metabolism
TMC4_092_endonuclease_VII_Shigella_phage_pSs-1	423	DNA, RNA and nucleotide metabolism
TMC4_239_DNA_ligase_Shigella_phage_pSs-1	114	DNA, RNA and nucleotide metabolism
TMC4_272_NrdA-like_aerobic_NDP_reductase_large_subunit_Shigella_phage_pSs-1	84	DNA, RNA and nucleotide metabolism
TMC4_002_DNA_topoisomerase_II_Shigella_phage_pSs-1	551	DNA, RNA and nucleotide metabolism
TMC4_004_Ndd-like_nucleoid_disruption_protein_Shigella_phage_pSs-1	1102	DNA, RNA and nucleotide metabolism
TMC4_017_DNA_topoisomerase_II_large_subunit_Shigella_phage_pSs-1	543	DNA, RNA and nucleotide metabolism
TMC4_029_Dda-like_helicase_Shigella_phage_pSs-1	325	DNA, RNA and nucleotide metabolism
TMC4_046_DNA_primase_Shigella_phage_pSs-1	47	DNA, RNA and nucleotide metabolism
TMC4_051_Dmd_discriminator_of_mRNA_degradation_Shigella_phage_pSs-1	2102	DNA, RNA and nucleotide metabolism
TMC4_057_thymidylate_synthase_Shigella_phage_pSs-1	160	DNA, RNA and nucleotide metabolism
TMC4_064_clamp_loader_of_DNA_polymerase_Shigella_phage_pSs-1	168	DNA, RNA and nucleotide metabolism
TMC4_068_SbcC-like_subunit_of_palindrome_specific_endonuclease_Shigella_phage_pSs-1	77	DNA, RNA and nucleotide metabolism
TMC4_168_DNA_end_protector_Shigella_phage_pSs-1	429	DNA, RNA and nucleotide metabolism
TMC4_213_DNA_helicase_Shigella_phage_pSs-1	16	DNA, RNA and nucleotide metabolism
TMC4_214_DNA_helicase_Shigella_phage_pSs-1	1143	DNA, RNA and nucleotide metabolism
TMC4_217_UvsY-like_recombination_mediator_Shigella_phage_pSs-1	231	DNA, RNA and nucleotide metabolism
TMC4_238_DNA_ligase_Shigella_phage_pSs-1	114	DNA, RNA and nucleotide metabolism
TMC4_270_endonuclease_Shigella_phage_pSs-1	1111	DNA, RNA and nucleotide metabolism
TMC4_276_thymidylate_synthase_Shigella_phage_pSs-1	160	DNA, RNA and nucleotide metabolism
TMC4_284_single_strand_DNA_binding_protein_Shigella_phage_pSs-1	224	DNA, RNA and nucleotide metabolism
TMC4_285_DNA_helicase_loader_Shigella_phage_pSs-1	269	DNA, RNA and nucleotide metabolism
TMC4_287_hypothetical_protein_Shigella_phage_pSs-1	107	DNA, RNA and nucleotide metabolism
TMC4_011_DenB-like_DNA_endonuclease_IV_Shigella_phage_pSs-1	1360	DNA, RNA and nucleotide metabolism
TMC4_032_RNA_polymerase_ADP-ribosylase_Shigella_phage_pSs-1	832	DNA, RNA and nucleotide metabolism
TMC4_033_RNA_polymerase_ADP-ribosylase_Shigella_phage_pSs-1	832	DNA, RNA and nucleotide metabolism
TMC4_034_RNA_polymerase_ADP-ribosylase_Shigella_phage_pSs-1	832	DNA, RNA and nucleotide metabolism
TMC4_035_RNA_polymerase_ADP-ribosylase_Shigella_phage_pSs-1	832	DNA, RNA and nucleotide metabolism
TMC4_059_DNA_polymerase_Shigella_phage_pSs-1	262	DNA, RNA and nucleotide metabolism
TMC4_060_DNA_polymerase_Shigella_phage_pSs-1	262	DNA, RNA and nucleotide metabolism
TMC4_063_clamp_loader_of_DNA_polymerase_Shigella_phage_pSs-1	225	DNA, RNA and nucleotide metabolism
TMC4_066_RNA_polymerase_binding_Shigella_phage_pSs-1	1285	DNA, RNA and nucleotide metabolism
TMC4_071_SbcD-like_subunit_of_palindrome_specific_endonuclease_Shigella_phage_pSs-1	100	DNA, RNA and nucleotide metabolism
TMC4_072_SbcD-like_subunit_of_palindrome_specific_endonuclease_Shigella_phage_pSs-1	100	DNA, RNA and nucleotide metabolism
TMC4_087_anaerobic_ribonucleotide_reductase_small_subunit_Shigella_phage_pSs-1	626	DNA, RNA and nucleotide metabolism
TMC4_088_anaerobic_ribonucleoside_reductase_large_subunit_Shigella_phage_pSs-1	4218	DNA, RNA and nucleotide metabolism
TMC4_091_endonuclease_VII_Shigella_phage_pSs-1	24351	DNA, RNA and nucleotide metabolism
TMC4_095_ribonucleotide_reductase_Shigella_phage_pSs-1	2294	DNA, RNA and nucleotide metabolism
TMC4_098_NrdC_thioredoxin_Shigella_phage_pSs-1	22	DNA, RNA and nucleotide metabolism
TMC4_110_hypothetical_protein	388	DNA, RNA and nucleotide metabolism
TMC4_128_valyl_tRNA_synthetase_modifier_Shigella_phage_pSs-1	1256	DNA, RNA and nucleotide metabolism
TMC4_130_endoribonuclease_Shigella_phage_pSs-1	1323	DNA, RNA and nucleotide metabolism
TMC4_164_RNA_ligase_Shigella_phage_pSs-1	755	DNA, RNA and nucleotide metabolism
TMC4_207_RNA_ligase_Shigella_phage_pSs-1	548	DNA, RNA and nucleotide metabolism
TMC4_208_RNA_ligase_Shigella_phage_pSs-1	548	DNA, RNA and nucleotide metabolism
TMC4_212_DNA_helicase_Shigella_phage_pSs-1	16	DNA, RNA and nucleotide metabolism
TMC4_271_ribonucleotide_reductase_class_Ia_beta_subunit_Shigella_phage_pSs-1	86	DNA, RNA and nucleotide metabolism
TMC4_273_NrdA-like_aerobic_NDP_reductase_large_subunit_Shigella_phage_pSs-1	3987	DNA, RNA and nucleotide metabolism
TMC4_277_thymidylate_synthase_Shigella_phage_pSs-1	160	DNA, RNA and nucleotide metabolism
TMC4_279_dihydrofolate_reductase_Shigella_phage_pSs-1	316	DNA, RNA and nucleotide metabolism
TMC4_163_internal_head_protein_Shigella_phage_pSs-1	3499	head and packaging
TMC4_194_terminase_small_subunit_Shigella_phage_pSs-1	735	head and packaging
TMC4_206_capsid_vertex_protein_Shigella_phage_pSs-1	138	head and packaging
TMC4_043_virion_structural_protein_Shigella_phage_pSs-1	1715	head and packaging
TMC4_196_terminase_large_subunit_Shigella_phage_pSs-1	2	head and packaging
TMC4_210_Hoc-like_head_decoration_Shigella_phage_pSs-1	1149	head and packaging
TMC4_053_head_vertex_assembly_chaperone_Shigella_phage_pSs-1	999	head and packaging
TMC4_137_internal_virion_protein_Shigella_phage_pSs-1	3155	head and packaging
TMC4_200_portal_protein_Shigella_phage_pSs-1	213	head and packaging
TMC4_203_head_maturation_protease_Shigella_phage_pSs-1	207	head and packaging
TMC4_204_head_scaffolding_protein_Shigella_phage_pSs-1	237	head and packaging
TMC4_205_major_head_protein_Shigella_phage_pSs-1	138	head and packaging
TMC4_211_minor_head_protein_inhibitor_of_protease_Shigella_phage_pSs-1	1051	head and packaging
TMC4_249_head_morphogenesis_Shigella_phage_pSs-1	931	head and packaging
TMC4_195_terminase_small_subunit_Shigella_phage_pSs-1	735	head and packaging
TMC4_201_hypothetical_protein	1064	head and packaging
TMC4_202_head_scaffolding_protein_Shigella_phage_pSs-1	1049	head and packaging
TMC4_121_lysis_inhibition_Shigella_phage_pSs-1	1246	lysis
TMC4_295_holin_Shigella_phage_pSs-1	860	lysis
TMC4_013_RIIB_lysis_inhibitor_Shigella_phage_pSs-1	609	lysis
TMC4_014_RIIA_lysis_inhibitor_Shigella_phage_pSs-1	612	lysis
TMC4_139_glycoside_hydrolase_family_protein_Shigella_phage_pSs-1	7	lysis
TMC4_248_lysis_inhibition;_accessory_protein_Shigella_phage_pSs-1	1457	lysis
TMC4_264_Rz-like_spanin_Shigella_phage_pSs-1	812	lysis
TMC4_265_Rz-like_spanin_Shigella_phage_pSs-1	739	lysis
TMC4_015_RIIA_lysis_inhibitor_Shigella_phage_pSs-1	612	lysis
TMC4_138_glycoside_hydrolase_family_protein_Shigella_phage_pSs-1	7	lysis
TMC4_233_Alt-like_RNA_polymerase_ADP-ribosyltransferase_Shigella_phage_pSs-1	802	moron, auxiliary metabolic gene and host takeover
TMC4_234_Alt-like_RNA_polymerase_ADP-ribosyltransferase_Shigella_phage_pSs-1	802	moron, auxiliary metabolic gene and host takeover
TMC4_096_antitoxin_from_a_toxin-antitoxin_system_Shigella_phage_pSs-1	3402	moron, auxiliary metabolic gene and host takeover
TMC4_111_hypothetical_protein_Shigella_phage_pSs-1	944	moron, auxiliary metabolic gene and host takeover
TMC4_141_hypothetical_protein_Shigella_phage_pSs-1	2203	moron, auxiliary metabolic gene and host takeover
TMC4_235_Alt-like_RNA_polymerase_ADP-ribosyltransferase_Shigella_phage_pSs-1	802	moron, auxiliary metabolic gene and host takeover
TMC4_021_cef_modifier_of_supressor_tRNAs_Shigella_phage_pSs-1	1354	moron, auxiliary metabolic gene and host takeover
TMC4_173_PAAR_motif_of_membran_proteins_Shigella_phage_pSs-1	281	moron, auxiliary metabolic gene and host takeover
TMC4_031_Srd_anti-sigma_factor_Shigella_phage_pSs-1	1400	moron, auxiliary metabolic gene and host takeover
TMC4_039_decoy_of_host_sigma32_Shigella_phage_pSs-1	2441	moron, auxiliary metabolic gene and host takeover
TMC4_090_anaerobic_ribonucleoside_reductase_large_subunit_Shigella_phage_pSs-1	487	moron, auxiliary metabolic gene and host takeover
TMC4_231_Alt-like_RNA_polymerase_ADP-ribosyltransferase_Shigella_phage_pSs-1	802	moron, auxiliary metabolic gene and host takeover
TMC4_232_Alt-like_RNA_polymerase_ADP-ribosyltransferase_Shigella_phage_pSs-1	802	moron, auxiliary metabolic gene and host takeover
TMC4_236_Alt-like_RNA_polymerase_ADP-ribosyltransferase_Shigella_phage_pSs-1	802	moron, auxiliary metabolic gene and host takeover
TMC4_056_beta-glucosyl-HMC-alpha-glucosyltransferase_Shigella_phage_pSs-1	842	other
TMC4_122_thymidine_kinase_Shigella_phage_pSs-1	592	other
TMC4_127_phosphatase_Shigella_phage_pSs-1	335	other
TMC4_140_nudix_hydrolase_Shigella_phage_pSs-1	1185	other
TMC4_260_hypothetical_protein	505	other
TMC4_007_periplasmic_protein_Shigella_phage_pSs-1	2401	other
TMC4_049_spackle_periplasmic_Shigella_phage_pSs-1	1586	other
TMC4_054_recombinase_Shigella_phage_pSs-1	97	other
TMC4_061_translation_repressor_Shigella_phage_pSs-1	242	other
TMC4_073_alpha-glucosyltransferase_Shigella_phage_pSs-1	2888	other
TMC4_009_hypothetical_protein	5011	other
TMC4_055_beta-glucosyl-HMC-alpha-glucosyltransferase_Shigella_phage_pSs-1	842	other
TMC4_094_inhibitor_of_host_Lon_protease_Shigella_phage_pSs-1	2167	other
TMC4_166_deoxynucleoside_monophosphate_kinase_Shigella_phage_pSs-1	139	other
TMC4_261_polynucleotide_kinase_Shigella_phage_pSs-1	505	other
TMC4_165_tail_fiber_chaperone_Shigella_phage_pSs-1	1002	tail
TMC4_174_baseplate_wedge_subunit_Shigella_phage_pSs-1	219	tail
TMC4_180_baseplate_wedge_subunit_Shigella_phage_pSs-1	977	tail
TMC4_199_tail_protein_Shigella_phage_pSs-1	45	tail
TMC4_290_hinge_connector_of_long_tail_fiber_protein_distal_connector_Shigella_phage_pSs-1	1425	tail
TMC4_291_long_tail_fiber_protein_distal_subunit_Shigella_phage_pSs-1	1699	tail
TMC4_167_tail_protein_Shigella_phage_pSs-1	45	tail
TMC4_176_baseplate_wedge_subunit_Shigella_phage_pSs-1	964	tail
TMC4_183_baseplate_wedge_subunit_Shigella_phage_pSs-1	958	tail
TMC4_184_baseplate_wedge_subunit_Shigella_phage_pSs-1	963	tail
TMC4_193_tail_sheath_stabilizer_Shigella_phage_pSs-1	227	tail
TMC4_227_baseplate_tail_tube_cap_Shigella_phage_pSs-1	230	tail
TMC4_170_baseplate_wedge_subunit_Shigella_phage_pSs-1	232	tail
TMC4_171_baseplate_hub_subunit_and_tail_lysozyme_Shigella_phage_pSs-1	430	tail
TMC4_177_baseplate_wedge_subunit_Shigella_phage_pSs-1	964	tail
TMC4_181_baseplate_wedge_tail_fiber_protein_connector_Shigella_phage_pSs-1	967	tail
TMC4_185_tail_collar_fiber_protein_Shigella_phage_pSs-1	910	tail
TMC4_218_baseplate_wedge_subunit_Shigella_phage_pSs-1	261	tail
TMC4_219_baseplate_hub_Shigella_phage_pSs-1	150	tail
TMC4_221_baseplate_hub_assembly_catalyst_Shigella_phage_pSs-1	150	tail
TMC4_222_baseplate_hub_Shigella_phage_pSs-1	1135	tail
TMC4_226_baseplate_hub_subunit_and_tail_length_Shigella_phage_pSs-1	1283	tail
TMC4_229_tail_tube_Shigella_phage_pSs-1	45	tail
TMC4_267_RNA_ligase_and_tail_fiber_protein_attachment_catalyst_Shigella_phage_pSs-1	562	tail
TMC4_289_long_tail_fiber_protein_proximal_connector_Shigella_phage_pSs-1	1154	tail
TMC4_294_tail_fiber_protein;_host_specificity_Shigella_phage_pSs-1	2056	tail
TMC4_175_baseplate_wedge_subunit_Shigella_phage_pSs-1	219	tail
TMC4_178_baseplate_wedge_subunit_Shigella_phage_pSs-1	964	tail
TMC4_179_baseplate_wedge_subunit_Shigella_phage_pSs-1	964	tail
TMC4_182_baseplate_wedge_subunit_Shigella_phage_pSs-1	958	tail
TMC4_186_tail_collar_fiber_protein_Shigella_phage_pSs-1	910	tail
TMC4_187_fibritin_neck_whisker_Shigella_phage_pSs-1	1056	tail
TMC4_192_tail_sheath_stabilizer_Shigella_phage_pSs-1	227	tail
TMC4_197_tail_sheath_Shigella_phage_pSs-1	23	tail
TMC4_220_baseplate_hub_Shigella_phage_pSs-1	150	tail
TMC4_223_baseplate_hub_Shigella_phage_pSs-1	1135	tail
TMC4_224_Baseplate_hub_assembly_protein_gp28	1140	tail
TMC4_225_baseplate_hub_distal_subunit_Shigella_phage_pSs-1	1140	tail
TMC4_228_baseplate_tail_tube_cap_Shigella_phage_pSs-1	230	tail
TMC4_268_RNA_ligase_and_tail_fiber_protein_attachment_catalyst_Shigella_phage_pSs-1	562	tail
TMC4_269_RNA_ligase_and_tail_fiber_protein_attachment_catalyst_Shigella_phage_pSs-1	562	tail
TMC4_288_tail_fiber_protein_proximal_subunit_Shigella_phage_pSs-1	972	tail
TMC4_292_long_tail_fiber_protein_distal_subunit_Shigella_phage_pSs-1	1699	tail
TMC4_293_long_tail_fiber_protein_distal_subunit_Shigella_phage_pSs-1	1699	tail
TMC4_022_MotB-like_transcriptional_regulator_Shigella_phage_pSs-1	1671	transcription regulation
TMC4_078_RNA_polymerase_sigma_factor_Shigella_phage_pSs-1	234	transcription regulation
TMC4_286_late_promoter_transcriptional_regulator_Shigella_phage_pSs-1	254	transcription regulation
TMC4_301_MotA-like_activator_of_middle_period_transcription_Shigella_phage_pSs-1	1345	transcription regulation
TMC4_037_Mrh_transcription_modulator_under_heat_shock_Shigella_phage_pSs-1	1234	transcription regulation
TMC4_040_Mrh_transcription_modulator_under_heat_shock_Shigella_phage_pSs-1	1234	transcription regulation
TMC4_120_starvation-inducible_transcriptional_regulator_Shigella_phage_pSs-1	858	transcription regulation
TMC4_266_inhibitor_of_host_transcription_Shigella_phage_pSs-1	1253	transcription regulation
TMC4_300_MotA-like_activator_of_middle_period_transcription_Shigella_phage_pSs-1	1345	transcription regulation
TMC4_250_SH3_beta-barrel_fold-containing_protein_Shigella_phage_pSs-1	1086	unknown function
TMC4_023_hypothetical_protein_Shigella_phage_pSs-1	2333	unknown function
TMC4_027_dextranase_Shigella_phage_pSs-1	1700	unknown function
TMC4_080_gp78_Shigella_phage_pSs-1	1475	unknown function
TMC4_082_gp80_Shigella_phage_pSs-1	2084	unknown function
TMC4_086_gp86_Shigella_phage_pSs-1	1930	unknown function
TMC4_124_hypothetical_protein_Shigella_phage_pSs-1	3452	unknown function
TMC4_134_autonomous_glycyl_radical_cofactor_GrcA_Shigella_phage_pSs-1	1209	unknown function
TMC4_136_hypothetical_protein_Shigella_phage_pSs-1	1488	unknown function
TMC4_143_hypothetical_protein_Shigella_phage_pSs-1	2604	unknown function
TMC4_243_hypothetical_protein_Shigella_phage_pSs-1	2042	unknown function
TMC4_244_hypothetical_protein_Shigella_phage_pSs-1	2153	unknown function
TMC4_254_hypothetical_protein_Shigella_phage_pSs-1	1504	unknown function
TMC4_263_hypothetical_protein_Shigella_phage_pSs-1	2347	unknown function
TMC4_274_hypothetical_protein	464	unknown function
TMC4_278_hypothetical_protein_Shigella_phage_pSs-1	9550	unknown function
TMC4_298_hypothetical_protein_Shigella_phage_pSs-1	839	unknown function
TMC4_016_gp17_Shigella_phage_pSs-1	1683	unknown function
TMC4_067_protein_GP45.2_Shigella_phage_pSs-1	1045	unknown function
TMC4_245_hypothetical_protein_Shigella_phage_pSs-1	1582	unknown function
TMC4_001_gp1_Shigella_phage_pSs-1	3487	unknown function
TMC4_005_hypothetical_protein_pSs1_006_Shigella_phage_pSs-1	2183	unknown function
TMC4_006_hypothetical_protein	4135	unknown function
TMC4_008_gp8_Shigella_phage_pSs-1	3098	unknown function
TMC4_010_gp11_Shigella_phage_pSs-1	2390	unknown function
TMC4_012_gp14_Shigella_phage_pSs-1	774	unknown function
TMC4_018_gp19_Shigella_phage_pSs-1	902	unknown function
TMC4_019_hypothetical_protein_Shigella_phage_pSs-1	622	unknown function
TMC4_020_hypothetical_protein	622	unknown function
TMC4_024_hypothetical_protein_Shigella_phage_pSs-1	1106	unknown function
TMC4_026_gp28_Shigella_phage_pSs-1	1700	unknown function
TMC4_028_gp30_Shigella_phage_pSs-1	4967	unknown function
TMC4_030_gp32_Shigella_phage_pSs-1	1079	unknown function
TMC4_036_gp36_Shigella_phage_pSs-1	1647	unknown function
TMC4_038_gp38_Shigella_phage_pSs-1	2681	unknown function
TMC4_041_gp41_Shigella_phage_pSs-1	2031	unknown function
TMC4_042_gp42_Shigella_phage_pSs-1	1873	unknown function
TMC4_045_gp45_Shigella_phage_pSs-1	2024	unknown function
TMC4_047_gp47_Shigella_phage_pSs-1	1823	unknown function
TMC4_048_gp48_Shigella_phage_pSs-1	4122	unknown function
TMC4_050_gp50_Shigella_phage_pSs-1	2375	unknown function
TMC4_058_gp59_Shigella_phage_pSs-1	1754	unknown function
TMC4_069_gp68_Shigella_phage_pSs-1	2327	unknown function
TMC4_070_gp69_Shigella_phage_pSs-1	2192	unknown function
TMC4_074_gp72_Shigella_phage_pSs-1	2140	unknown function
TMC4_075_gp73_Shigella_phage_pSs-1	1648	unknown function
TMC4_076_a-gt.4_family_protein_Shigella_phage_pSs-1	951	unknown function
TMC4_077_hypothetical_protein	1169	unknown function
TMC4_079_gp77_Shigella_phage_pSs-1	1822	unknown function
TMC4_081_gp79_Shigella_phage_pSs-1	1475	unknown function
TMC4_083_gp81_Shigella_phage_pSs-1	24953	unknown function
TMC4_084_gp82_Shigella_phage_pSs-1	1774	unknown function
TMC4_085_gp83_Shigella_phage_pSs-1	2283	unknown function
TMC4_093_gp91_Shigella_phage_pSs-1	3272	unknown function
TMC4_097_gp96_Shigella_phage_pSs-1	2112	unknown function
TMC4_099_hypothetical_protein_Shigella_phage_pSs-1	2052	unknown function
TMC4_100_hypothetical_protein_Shigella_phage_pSs-1	1474	unknown function
TMC4_101_hypothetical_protein_Shigella_phage_pSs-1	1435	unknown function
TMC4_102_hypothetical_protein_Shigella_phage_pSs-1	1435	unknown function
TMC4_103_hypothetical_protein_Shigella_phage_pSs-1	2351	unknown function
TMC4_104_hypothetical_protein_Shigella_phage_pSs-1	2257	unknown function
TMC4_105_hypothetical_protein_Shigella_phage_pSs-1	2547	unknown function
TMC4_106_hypothetical_protein_Shigella_phage_pSs-1	2274	unknown function
TMC4_107_hypothetical_protein_Shigella_phage_pSs-1	2274	unknown function
TMC4_108_hypothetical_protein_Shigella_phage_pSs-1	1931	unknown function
TMC4_109_hypothetical_protein_Shigella_phage_pSs-1	1250	unknown function
TMC4_112_hypothetical_protein_Shigella_phage_pSs-1	1502	unknown function
TMC4_113_molybdopterin-guanine_dinucleotide_biosynthesis_protein_MobD_Shigella_phage_pSs-1	1502	unknown function
TMC4_114_hypothetical_protein_Shigella_phage_pSs-1	3215	unknown function
TMC4_115_hypothetical_protein_Shigella_phage_pSs-1	3515	unknown function
TMC4_116_hypothetical_protein_Shigella_phage_pSs-1	2758	unknown function
TMC4_117_hypothetical_protein_Shigella_phage_pSs-1	653	unknown function
TMC4_118_hypothetical_protein_Shigella_phage_pSs-1	653	unknown function
TMC4_119_hypothetical_protein_Shigella_phage_pSs-1	3024	unknown function
TMC4_123_hypothetical_protein_Shigella_phage_pSs-1	653	unknown function
TMC4_125_hypothetical_protein_Shigella_phage_pSs-1	2306	unknown function
TMC4_126_hypothetical_protein_Shigella_phage_pSs-1	2008	unknown function
TMC4_129_hypothetical_protein_Shigella_phage_pSs-1	600	unknown function
TMC4_131_hypothetical_protein_Shigella_phage_pSs-1	2037	unknown function
TMC4_132_hypothetical_protein_Shigella_phage_pSs-1	717	unknown function
TMC4_133_hypothetical_protein_Shigella_phage_pSs-1	1986	unknown function
TMC4_135_hypothetical_protein_Shigella_phage_pSs-1	2049	unknown function
TMC4_142_hypothetical_protein_Shigella_phage_pSs-1	2330	unknown function
TMC4_144_hypothetical_protein_Shigella_phage_pSs-1	2607	unknown function
TMC4_145_hypothetical_protein_Shigella_phage_pSs-1	1203	unknown function
TMC4_146_hypothetical_protein_Shigella_phage_pSs-1	6869	unknown function
TMC4_147_hypothetical_protein_Shigella_phage_pSs-1	2678	unknown function
TMC4_148_hypothetical_protein_Shigella_phage_pSs-1	2280	unknown function
TMC4_149_hypothetical_protein_Shigella_phage_pSs-1	2280	unknown function
TMC4_160_hypothetical_protein_Shigella_phage_pSs-1	2023	unknown function
TMC4_161_hypothetical_protein_Shigella_phage_pSs-1	1411	unknown function
TMC4_162_hypothetical_protein_Shigella_phage_pSs-1	1692	unknown function
TMC4_172_hypothetical_protein_Shigella_phage_pSs-1	1168	unknown function
TMC4_198_hypothetical_protein_Shigella_phage_pSs-1	5104	unknown function
TMC4_209_hypothetical_protein	1566	unknown function
TMC4_215_hypothetical_protein_Shigella_phage_pSs-1	1043	unknown function
TMC4_216_hypothetical_protein_Shigella_phage_pSs-1	1541	unknown function
TMC4_230_hypothetical_protein_Shigella_phage_pSs-1	1739	unknown function
TMC4_237_hypothetical_protein_Shigella_phage_pSs-1	1976	unknown function
TMC4_240_hypothetical_protein	2054	unknown function
TMC4_241_hypothetical_protein_Shigella_phage_pSs-1	420	unknown function
TMC4_242_hypothetical_protein_Shigella_phage_pSs-1	652	unknown function
TMC4_246_hypothetical_protein_Shigella_phage_pSs-1	1507	unknown function
TMC4_247_hypothetical_protein	1427	unknown function
TMC4_251_hypothetical_protein_Shigella_phage_pSs-1	2010	unknown function
TMC4_253_hypothetical_protein_Shigella_phage_pSs-1	2063	unknown function
TMC4_255_hypothetical_protein_Shigella_phage_pSs-1	3710	unknown function
TMC4_256_hypothetical_protein_Shigella_phage_pSs-1	3710	unknown function
TMC4_257_hypothetical_protein_Shigella_phage_pSs-1	1695	unknown function
TMC4_258_hypothetical_protein	2360	unknown function
TMC4_259_hypothetical_protein_Shigella_phage_pSs-1	2430	unknown function
TMC4_262_hypothetical_protein_Shigella_phage_pSs-1	2364	unknown function
TMC4_275_hypothetical_protein_Shigella_phage_pSs-1	2458	unknown function
TMC4_280_hypothetical_protein_Shigella_phage_pSs-1	3183	unknown function
TMC4_281_hypothetical_protein	1254	unknown function
TMC4_282_hypothetical_protein_Shigella_phage_pSs-1	622	unknown function
TMC4_283_hypothetical_protein_Shigella_phage_pSs-1	1764	unknown function
TMC4_296_hypothetical_protein_Shigella_phage_pSs-1	1359	unknown function
TMC4_297_hypothetical_protein_Shigella_phage_pSs-1	1844	unknown function
TMC4_299_hypothetical_protein_Shigella_phage_pSs-1	1594	unknown function

## References

[1] Hussen S (2019). Annals of clinical microbiology and antimicrobials..

[2] Morozoff C (2024). Open forum infectious diseases..

[3] Kotloff K.L (1999). Bulletin of the World Health Organization..

[4] Ranjbar R, Abbas F (2019). Infection and drug resistance..

[5] Derek M (2017). World journal of gastrointestinal pharmacology and therapeutics..

[6] Baker S, Scott AT (2023). Microbiology..

[7] Marzanna S (2022). Journal of biomedical science..

[8] Lynn H.E (2019). Clinical infectious diseases..

[9] Anandhalakshmi S (2024). Frontiers in microbiology..

[10] Sabrina R (2021). Archives of microbiology..

[11] Fujiki J, Bernd S (2023). JHEP reports: innovation in hepatology..

[12] Shahin K, Majid B (2018). Journal of food science and technology..

[13] Ahamed S.K.T (2023). Frontiers in microbiology..

[14] Yang F (2005). Nucleic acids research..

[15] Klimenko A.I (2016). BMC microbiology..

[16] Subramanian S (2020). Annual review of virology..

[17] Gao Z, Yue F (2023). Frontiers in microbiology..

[18] Oromí-Bosch A (2023). Annual review of virology..

[19] Borin J.M (2021). Proceedings of the National Academy of Sciences of the United States of America..

[20] Dover J.A (2016). Genome biology and evolution..

[21] Watson B.N.J (2023). PLoS biology..

[22] The H.C (2016). Nature reviews. Microbiology..

[23] Rossi F.P.N (2024). Methods in molecular biology (Clifton, N.J.)..

[24] Ceballos-Garzon A (2022). Pathogens and disease..

[25] Marino N.D (2020). Nature methods..

[26] Zhang Y (2022). Frontiers in microbiology..

[27] https://www.bioinformatics.babraham.ac.uk/projects/fastqc/.

[28] Bolger A.M (2014). Bioinformatics..

[29] Li D (2016). Methods.

[30] https://bioinformaticshome.com/tools/wga/descriptions/Velvet.html.

[31] Prjibelski A (2020). Current protocols in bioinformatics..

[32] Souvorov A (2018). Genome biology..

[33] Gurevich A (2013). Bioinformatics..

[34] Seemann T (2014). Bioinformatics..

[35] Terzian P (2021). NAR genomics and bioinformatics..

[36] https://blast.ncbi.nlm.nih.gov/Blast.cgi?PAGE=Proteins.

[37] Page A.J (2015). Bioinformatics..

[38] https://anaconda.org/bioconda/codonw.

[39] Stamatakis A (2014). Bioinformatics..

[40] Huang L (2021). Nucleic acids research..

[41] Dong C (2018). Nucleic acids research..

[42] Alcock B.P (2023). Nucleic acids research..

[43] Grant J.R (2023). Nucleic acids research..

[44] Van den Berg D.F (2023). ELife..

[45] Ahamed S.T (2019). Frontiers in microbiology..

[46] Prevelige P.E, Jr Juilana R.C (2018). Current opinion in virology..

[47] Shymialevich D (2024). International journal of molecular sciences..

[48] Naureen Z (2020). Acta bio-medica: Atenei Parmensis..

[49] Silva M.D (2024). mSystems..

[50] Borin J.M (2021). Proceedings of the National Academy of Sciences of the United States of America..

[51] Wolput S (2024). Nucleic acids research..

[52] Stone E (2019). Viruses..

[53] Wróbel A (2020). The Journal of antibiotics..

